# Global trends and emerging frontiers in cancer immunotherapy and gut microbiota-derived metabolites: a bibliometric analysis

**DOI:** 10.3389/fcimb.2026.1876450

**Published:** 2026-09-03

**Authors:** Yingying Hu, Chenhuai Teng, Dian Zhang

**Affiliations:** 1 Department of Gastroenterology, Sir Run Run Shaw Hospital, School of Medicine, Zhejiang University, Hangzhou, Zhejiang, China; 2 Department of Emergency Medicine, the Second Affiliated Hospital of Wenzhou Medical University, Wenzhou, China; 3 School of Medicine, Shaoxing University, Shaoxing, China

**Keywords:** bibliometrics, cancer immunotherapy, gut microbiota, intratumoral microbiota, microbial metabolites, gut-liver axis

## Abstract

**Objectives:**

Cancer immunotherapy has achieved remarkable clinical success; however, therapeutic resistance remains a major challenge. Increasing evidence suggests that gut microbiota-derived metabolites play a critical role in modulating host immune responses and influencing treatment outcomes. This study aimed to systematically characterize the global research landscape and identify emerging trends in this field using bibliometric approaches.

**Methods:**

Publications were retrieved from the Web of Science Core Collection (WoSCC) and Scopus databases based on predefined search strategies. Only English-language articles and reviews were included. Bibliographic records were integrated and deduplicated using the bibliometrix R package. Descriptive analyses were performed to evaluate publication outputs, countries, institutions, authors, and journals. CiteSpace was used for reference burst detection, and VOSviewer was applied to construct keyword co-occurrence networks.

**Results:**

A total of 2,979 publications from 1973 to March 2026 were included, comprising 1,593 reviews and 1,386 original articles. China contributed the largest number of publications (1,121; 37.63%), followed by the United States (499; 16.75%). Notably, the United States exhibited the highest H-index and centrality, indicating its leading role in global research impact and collaboration networks. Sichuan University was the most productive institution (115 publications), with six of the top ten institutions located in China. Laurence Zitvogel (28 publications) and Guido Kroemer (22 publications) were the leading authors in this field. Frontiers in Immunology published the highest number of relevant articles (277). Keyword and temporal analyses indicated a shift from descriptive microbiome profiling toward mechanism-oriented and translational research. Recent hotspots include “immunomodulation”, “the gut–liver axis”, “intratumoral microbiota”, and “microbial metabolites”.

**Conclusions:**

Microbial metabolites have emerged as key functional mediators shaping responses to cancer immunotherapy, reflecting a shift from microbiome composition toward metabolite-centered mechanisms. This field is undergoing a transition from descriptive profiling to mechanism-oriented and translational research. These findings provide important insights for developing microbiota-targeted strategies to enhance the efficacy of cancer immunotherapy.

## 1 Introduction

Immunotherapy has emerged as a pivotal therapeutic strategy in oncology by activating the host immune system to recognize and eliminate cancer cells, thereby exerting durable antitumor effects (Yang et al., 2023; Yasinjan et al., 2023). Among these approaches, immune checkpoint inhibitors (ICIs) represent one of the most successful classes of immunotherapeutic agents (Li et al., 2019). By blocking inhibitory immune checkpoints, such as programmed cell death protein 1/programmed death-ligand 1 (PD-1/PD-L1) and cytotoxic T-lymphocyte-associated antigen 4 (CTLA-4), ICIs restore antitumor immune responses and counteract tumor immune evasion (Arafat Hossain, 2024; Roerden and Spranger, 2025). In parallel, chimeric antigen receptor (CAR)-based therapies have further expanded the scope of cancer immunotherapy. These strategies involve the genetic engineering of immune cells, including T cells, macrophages, and NK cells, to generate CAR-T, CAR-macrophage, and CAR-NK cells. These engineered cells enable targeted recognition and elimination of tumor-associated antigens (Labanieh et al., 2018; Hu and Huang, 2020; Xie et al., 2020; Baker et al., 2023; Wala and Hanna, 2023; Wang et al., 2024). Despite these advances, a substantial proportion of patients fail to respond or eventually develop resistance to immunotherapy, which remains a major clinical challenge (Conforti et al., 2018; Vesely et al., 2022; Lefler et al., 2024). For example, primary and acquired resistance to immune checkpoint inhibitors has been widely reported, with only a subset of patients achieving durable clinical responses (Conforti et al., 2018). These observations underscore the urgent need to identify novel modulators and predictive factors that influence immunotherapy efficacy.

In recent years, the gut microbiota has been increasingly recognized as a key regulator of host immunity and disease progression (Chen et al., 2021; Christovich and Luo, 2022). Accumulating evidence indicates that gut microbiota composition is closely associated with the efficacy of cancer immunotherapy (Zhou et al., 2021; Li et al., 2024). For example, a phase Ib/II clinical trial demonstrated that colorectal cancer patients with a higher abundance of Fusobacterium exhibited significantly poorer responses to combined regorafenib and toripalimab therapy, accompanied by reduced progression-free survival (Wang et al., 2021). However, taxonomic composition alone cannot fully explain the complex interactions between the gut microbiota and host immunity. Increasing evidence indicates that these interactions are largely mediated by the functional output of the microbiota, particularly gut microbiota-derived metabolites. Clinical studies have shown that metabolomic profiles, including amino acid and lipid metabolism, are significantly associated with favorable responses to immunotherapy (Rosario et al., 2024). In addition, key gut microbiota-derived metabolites, such as short-chain fatty acids (SCFAs), tryptophan metabolites, and secondary bile acids, have been widely reported to regulate immune cell function, cytokine production, and inflammatory responses (Rooks and Garrett, 2016; Ala, 2022; Fiorucci et al., 2026). For instance, SCFAs, especially butyrate, exert immunomodulatory effects by influencing intestinal epithelial cells, innate immune cells, and T cell differentiation (Mann et al., 2024). Consistently, emerging clinical evidence suggests that higher levels of SCFAs or enrichment of SCFA-producing bacteria are associated with improved responses to ICIs in patients with cancer, supporting their potential role in enhancing antitumor immunity (Yang et al., 2025).

Despite the growing body of literature, studies in this field remain fragmented, with most investigations focusing on either microbial composition or individual mechanistic pathways. A comprehensive and systematic evaluation of global research trends, particularly focusing on gut microbiota-derived metabolites in cancer immunotherapy, is still lacking. Bibliometric analysis provides a quantitative framework for assessing large-scale scientific literature, enabling the identification of research hotspots, knowledge structures, and emerging trends (Wang et al., 2024). Therefore, this study systematically investigated the research landscape of cancer immunotherapy and gut microbiota-derived metabolites using a comprehensive bibliometric approach. Publications retrieved from the Web of Science Core Collection (WoSCC) and Scopus databases were analyzed using Bibliometrix, CiteSpace, and VOSviewer (Li et al., 2024). This study seeks to provide a structured overview of current research developments and to offer insights into future directions and potential translational applications in this rapidly evolving field.

## 2 Materials and methods

### 2.1 Study design

This study was designed as a bibliometric analysis to systematically evaluate global research trends, collaboration patterns, and emerging hotspots in the field of cancer immunotherapy and gut microbiota metabolism. The study was conducted in accordance with a predefined search strategy and analytical workflow using established bibliometric tools, including Bibliometrix, CiteSpace, and VOSviewer.

### 2.2 Data sources and search strategy

The literature data were retrieved from the Web of Science Core Collection (WoSCC) and Scopus databases, which were selected because they provide comprehensive multidisciplinary coverage, extensive citation indexing, and are among the most widely used databases for bibliometric analyses. No restrictions were imposed on publication year. The final search was conducted on March 10, 2026.

The search strategy was developed using predefined keywords related to cancer immunotherapy, malignancies, gut microbiota, and microbial metabolism. In WoSCC, the search was conducted using the “Topic” field, whereas in Scopus, the corresponding strategy was applied using the “TITLE-ABS-KEY” field. The same conceptual keyword combinations were used across both databases, with only database-specific syntax modifications. The complete search strategies for WoSCC and Scopus are provided in **Supplementary Table 1**.

Broad metabolite-related terms were intentionally included to maximize retrieval sensitivity and capture emerging studies investigating microbiota-associated metabolic mechanisms, including studies focusing on metabolomic profiles or newly identified microbial metabolites. All records were exported in plain text format, including full bibliographic information and cited references, for subsequent screening and bibliometric analyses.

### 2.3 Study selection and eligibility criteria

Only English-language publications classified as original articles or reviews were included. Literature screening was independently performed by three researchers.

The exclusion criteria were as follows: Conference papers, editorials, commentaries, letters, and other non-article publication types; Studies without a clear relationship to cancer immunotherapy and gut microbiota-associated metabolic mechanisms; Duplicate records identified across databases.

Data retrieval, study screening, and data extraction were independently conducted by two authors, followed by cross-verification. Any disagreements were resolved through discussion with a third author until consensus was reached. Inter-reviewer agreement was assessed and demonstrated excellent consistency.

Records retrieved from WoSCC and Scopus were merged and deduplicated using the “mergeDbSources” function implemented in the Bibliometrix R package. Subsequently, titles and abstracts were manually screened to verify thematic relevance. The screening process is shown in **Supplementary Table 2**. Publications were considered relevant when they investigated the relationship between cancer immunotherapy and gut microbiota-associated metabolic mechanisms, including microbiota-derived metabolites, metabolomic alterations, or metabolic pathways involved in immune regulation. Records without a clear connection to these topics were excluded. When relevance could not be determined from titles and abstracts, additional full-text assessment was performed.

### 2.4 Data extraction

The following bibliographic information was extracted from each included publication: title, authors, affiliations, country/region, journal, publication year, keywords, references, citation counts, and document type. These data were used to evaluate publication trends, contributions of countries and institutions, author productivity, collaboration networks, keyword co-occurrence, and reference citation patterns.

To further evaluate the extent to which the retrieved literature focused on microbial metabolites, an additional keyword-based classification was performed among original research articles using predefined metabolite-related terms derived from the original search strategy. Titles, abstracts, and author keywords were screened for these predefined terms, covering bile acids, short-chain fatty acids, tryptophan metabolites, vitamins, amino acids, amines, phenols, indoles, and other metabolite-related categories. Original articles containing at least one predefined metabolite-related term were classified as metabolite-related studies. The complete list of search terms is provided in the original search strategy (**Supplementary Table 1**).

### 2.5 Data analysis

Descriptive statistical analyses were conducted using R software (Version 4.5.1; R Project for Statistical Computing). Comprehensive bibliometric analyses were performed using the Bibliometrix R package (Version 4.3.2), including annual publication trends, country and institutional contributions, author productivity, keyword analysis, and reference analysis. Because review articles constituted a substantial proportion of the corpus, a targeted sensitivity analysis restricted to original articles was additionally performed to assess the robustness of the principal findings potentially influenced by document type. The sensitivity analysis focused on country-level contributions, institutional and journal productivity, and the major keyword, thematic, and temporal patterns identified through keyword co-occurrence, clustering, and timeline analyses. These analyses were selected to evaluate whether the main research landscape and thematic conclusions remained robust after excluding review articles.

CiteSpace (Version 6.4.R1) was used to construct collaboration networks among countries, institutions, and authors, and to perform burst detection analyses of keywords and references. The main parameters were set as follows: time slicing = 1 year, node types included Country, Institution, and Keyword, and selection criterion = Top 25. Betweenness centrality was calculated to assess the relative importance of nodes within collaboration networks. VOSviewer was used to visualize collaboration networks and keyword co-occurrence patterns. For keyword analysis, synonyms and spelling variants were harmonized using a predefined thesaurus file before analysis (**Supplementary Table 3**). VOSviewer and CiteSpace were used with their default settings, without additional parameter adjustments beyond the settings explicitly described in the Methods.

### 2.6 Ethical considerations

All data analyzed in this study were obtained from publicly accessible academic databases (Web of Science and Scopus). Therefore, ethical approval from an institutional review board was not required.

## 3 Results

### 3.1 Data fundamentals and annual publication trends

**Figure 1:**
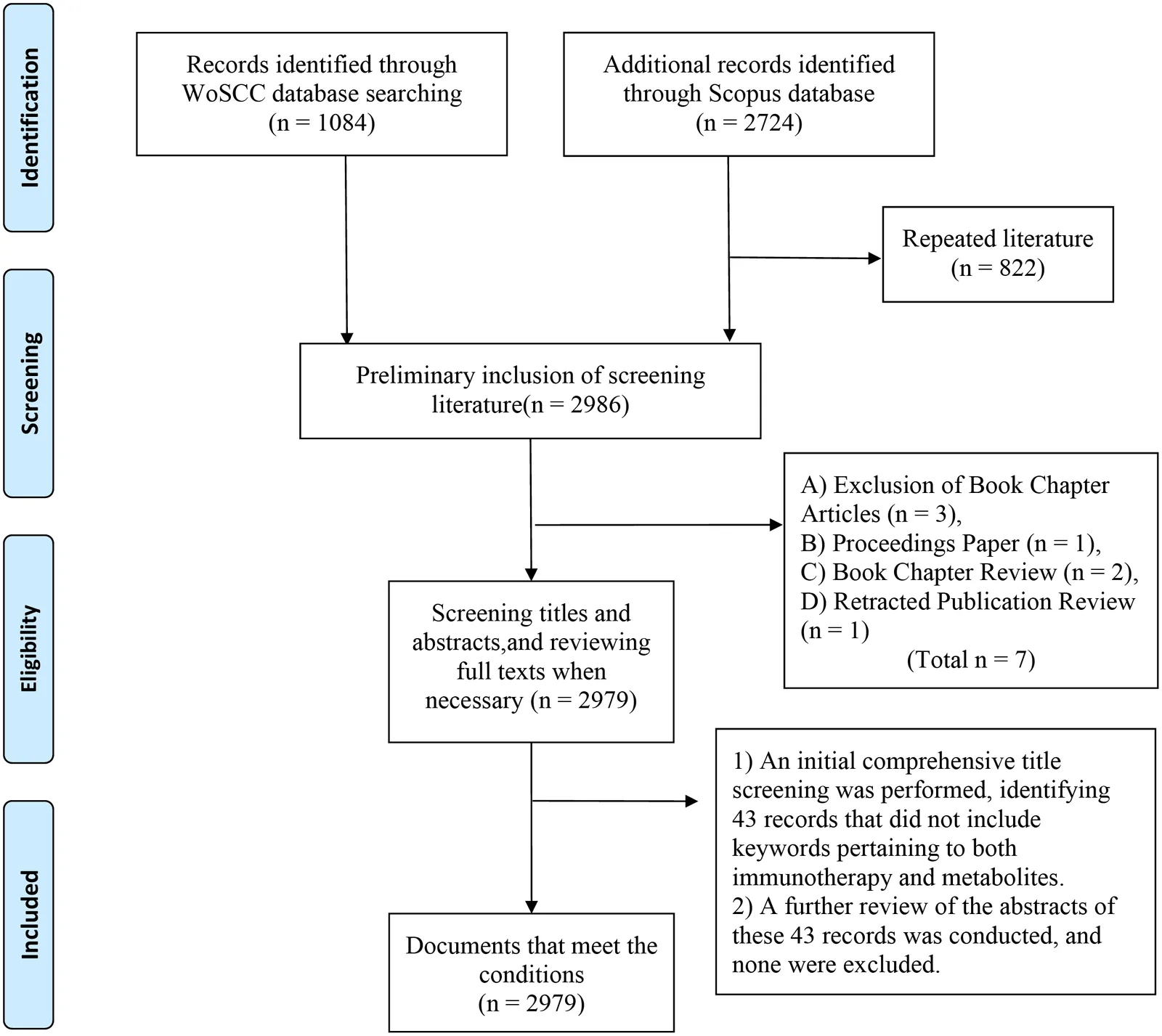
PRISMA flow diagram.

Following the initial literature search, a total of 1,084 and 2,724 records were retrieved from the WoSCC and Scopus databases, respectively, and imported into EndNote 20 for further processing. After excluding non-eligible document types (including letters, conference abstracts, editorials, proceedings papers, corrections, news items, retracted publications, and books) and restricting the language to English, a total of 2,979 publications were retained after database integration and deduplication, comprising 1,593 reviews and 1,386 original articles (including early access), as summarized in the PRISMA flow diagram (**Figure 1**) and **Table 1**. Among the original articles, 937 studies (67.60%) were classified as metabolite-related by applying the metabolite-related terms from the original search strategy to the title, abstract, and author keyword fields. These terms identified studies explicitly referring to metabolites or metabolite-related mechanisms. The identified studies included research on bile acids (n = 60), short-chain fatty acids (n = 160), acetate (n = 42), propionate (n = 13), neurotransmitters (n = 61), tryptophan metabolites (n = 54), vitamins (n = 61), amino acids (n = 204), amines (n = 175), phenols (n = 15), indoles (n = 62), and other metabolites (n = 50). This finding further supports the close relevance of the retrieved literature to microbial metabolism, although additional studies may have addressed metabolism-related mechanisms using broader terms such as “metabolism” or “metabolite” without specifying individual metabolites.

**Table 1:** Main information about the collection.

Description	Result
Time span	1973-2026
Sources (Journals,Books,etc.)	871
Documents	2979
Articles	1377
Articles: early access	9
Reviews	1585
Reviews: early access	8
Annual Growth Rate	10.14%
Authors	13101
Authors of single-authored docs	72
International Co-Authorship	6.579%
Co-Authors per Doc	7.74
Author’s Keywords (DE)	6269
References	536495

**Figure 2:**
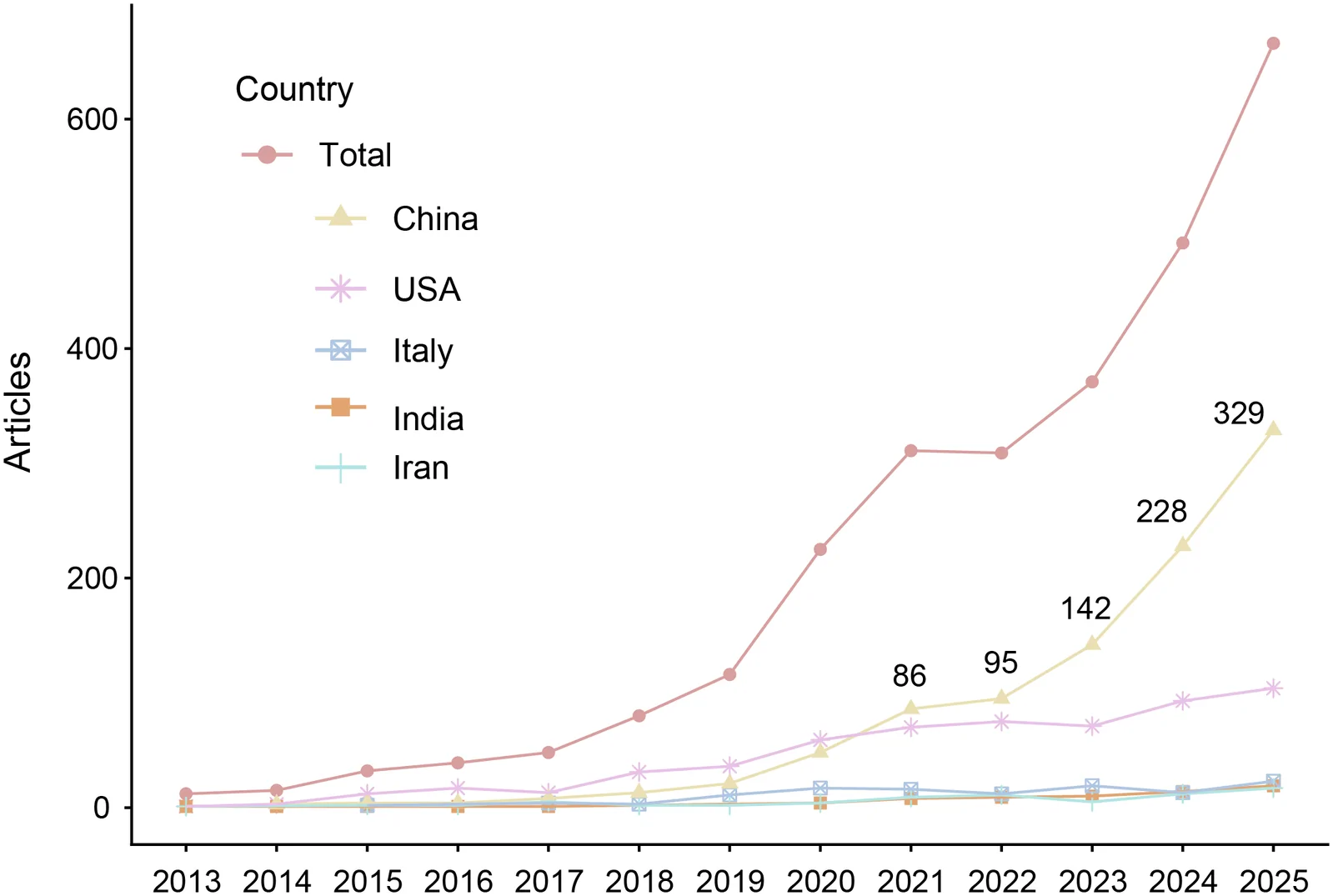
Literature change trends in the total number of publications and the top five countries with the largest number of publications from 2013 to 2025.

The annual publication trend from 1973 to 2025 demonstrated a steady increase, with an average annual growth rate of 10.14%. Between 1973 and 2013, fewer than 10 publications were produced annually, indicating a prolonged early stage of development. From 2013 to 2019, publication output increased gradually, followed by a marked and rapid growth after 2019 (**Figure 2**). As of the latest search date in 2026, a total of 167 publications had already been published.

### 3.2 Country-level contributions

**Figure 3:**
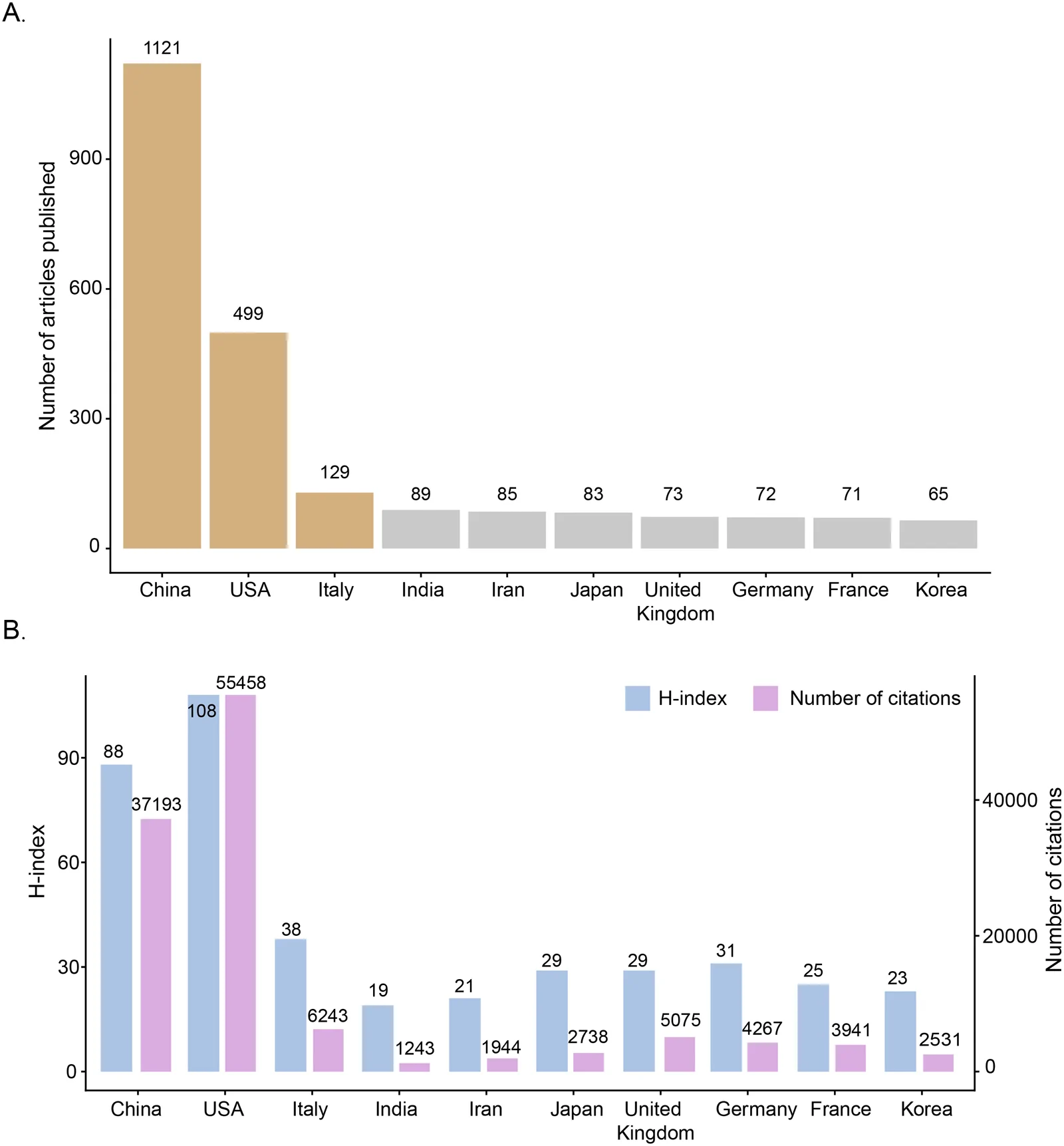
Research productivity and academic impact of the top ten most productive countries. **(A)** Number of publications by country. **(B)** H-index and total citations of the top ten countries.

A total of 75 countries/regions contributed to this research field. China ranked first with 1,121 publications (37.63%), followed by the United States (499, 16.75%), Italy (129, 4.33%), India (89, 2.98%), and Iran (85, 2.85%) (**Figure 3A**). Notably, China surpassed the United States in annual publication output after 2021 and has maintained a consistent upward trend, with 329 publications in 2025 and 94 publications recorded in 2026 to date (**Figure 2**).

The H-index is a composite metric that reflects both the productivity and academic impact of scientific output. Among the top 10 most productive countries, the United States exhibited the highest H-index and total citation count (H-index = 108; citations = 55,458), followed by China (H-index = 88; citations = 37,193) and Italy (H-index = 38; citations = 6,243) (**Figure 3B**), indicating that the United States holds a leading position in both research productivity and academic impact in this field. Sensitivity analysis restricted to original articles showed that China and the United States remained the leading contributors, with 562 and 245 publications, respectively, and H-index values of 54 and 71, respectively (**Supplementary Table 4**). Although publication and citation counts decreased after excluding reviews, the overall country-level pattern remained consistent.

**Figure 4:**
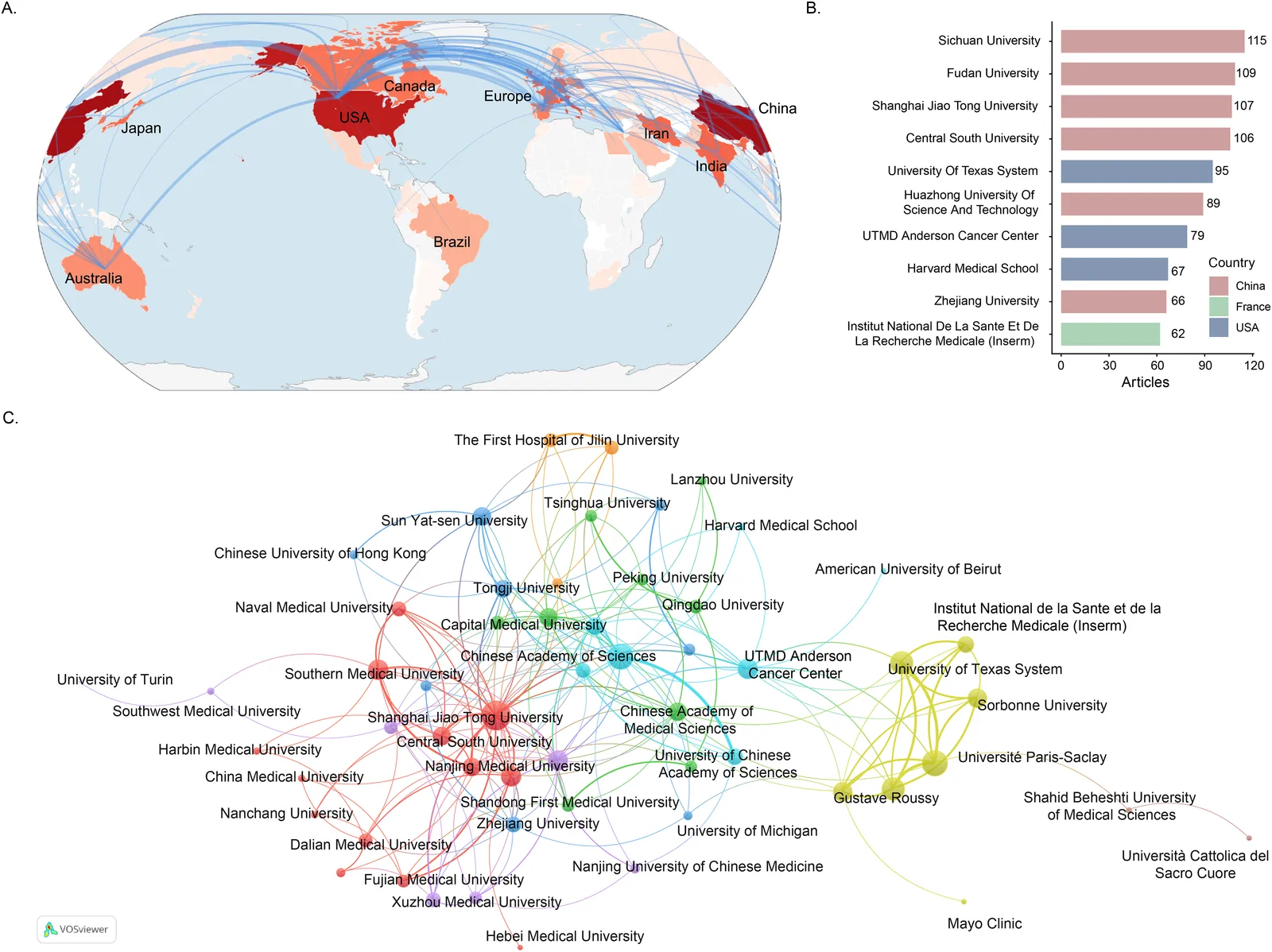
Global and institutional research landscape of cancer immunotherapy and gut microbiota metabolism. **(A)** National collaboration network; **(B)** Top ten most productive institutions; **(C)** Institutional collaboration network.

The country-level collaboration network demonstrated relatively strong international cooperation (**Figure 4A**). The United States occupied the most central position (centrality = 0.67), followed by Iran (0.26) and the United Kingdom (0.22), whereas China ranked 15th (0.04) (**Supplementary Table 5**).

### 3.3 Institutional landscape

At the institutional level (**Figure 4B**), the most productive institutions were Sichuan University (115 publications), Fudan University (109), and Shanghai Jiao Tong University (107), all from China. Notably, among the top 10 institutions, six were from China, three from the United States, and one from France (French National Institute of Health and Medical Research, INSERM). To further evaluate the contribution of primary research, an additional analysis restricted to original articles was performed. Under this criterion, Shanghai Jiao Tong University ranked first (40 articles), followed by Fudan University (33 articles) and the Chinese Academy of Sciences (23 articles), whereas Harvard Medical School and Huazhong University of Science and Technology each contributed 20 original articles (**Supplementary Table 6**). These findings suggest that institutional rankings may vary depending on document type, highlighting differences between overall academic output and primary research productivity. The most central institutions included the University of Texas System (0.13), The University of Texas MD Anderson Cancer Center (0.12), and the Institut National de la Santé et de la Recherche Médicale (INSERM) (0.08), indicating their prominent roles in global collaboration networks (**Figure 4C**).

### 3.4 Author contributions

**Figure 5:**
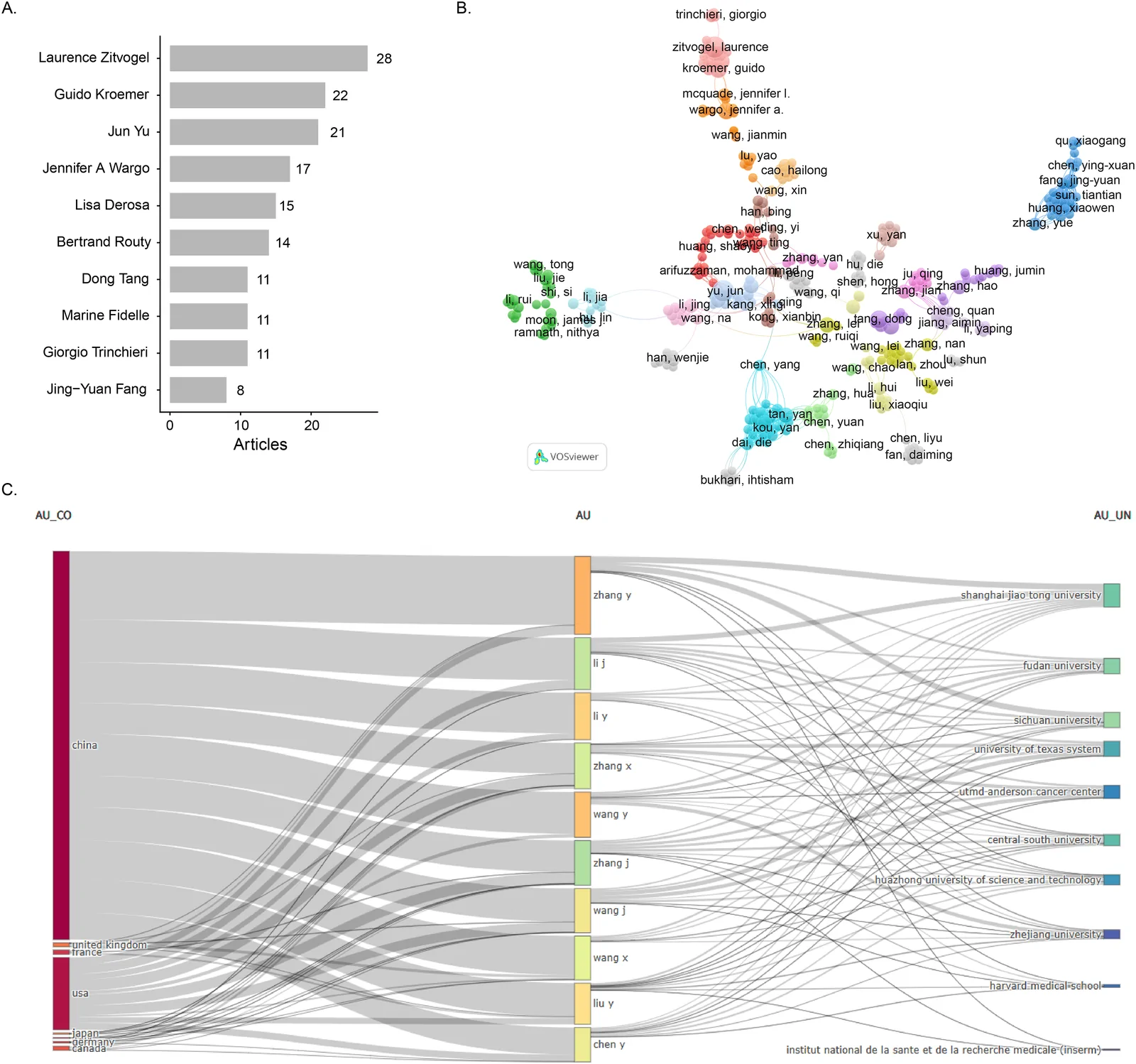
Author productivity and collaboration patterns in cancer immunotherapy and gut microbiota metabolism research. **(A)** Top ten most productive authors. **(B)** Author collaboration network. **(C)** Sankey diagram illustrating relationships among countries, institutions, and authors.

In terms of author productivity, Laurence Zitvogel (28 publications) and Guido Kroemer (22 publications), both affiliated with INSERM, were the leading contributors, followed by Jun Yu (21 publications) from the Chinese University of Hong Kong (**Figure 5A**). Notably, a large number of international researchers collaboratively contributed to the observational study titled “Dietary fiber and probiotics influence the gut microbiome and melanoma immunotherapy response”, published in Science in 2021 (Spencer et al., 2021).

The author collaboration network showed that Chinese researchers maintain relatively close cooperative relationships (**Figure 5B**).

Furthermore, the Sankey diagram illustrates the relationships among countries, institutions, and authors, revealing clear linkages between major contributing countries, leading institutions, and highly productive authors (**Figure 5C**).

### 3.5 Journals analysis

A total of 131 journals published at least five articles, contributing 1,818 publications (61.03% of the total output). Among the top 10 most productive journals (**Table 2**), Frontiers in Immunology published the highest number of articles (277), followed by International Journal of Molecular Sciences (89) and Cancers (73). All top 10 journals were classified as Q1 journals, with Seminars in Cancer Biology having the highest impact factor (20.3).

**Table 2:** The top ten journals with the most articles related to cancer immunotherapy and intestinal flora metabolism.

Journal	Nation	Number of articles published	Impact factor (2025-2026)	H-index	JCR
*Frontiers in Immunology*	Switzerland	277	7.0	84	Q1
*International Journal of Molecular Sciences*	USA	89	5.6	114	Q1
*Cancers*	Switzerland	73	4.8	53	Q1
*Frontiers in Oncology*	Switzerland	59	3.4	60	Q1
*Journal for ImmunoTherapy of Cancer*	USA	54	11.7	36	Q1
*Gut Microbes*	USA	35	15.3	–	Q1
*Frontiers in Microbiology*	Switzerland	32	5.8	88	Q1
*Cells*	Switzerland	31	6.0	14	Q1
*Seminars in Cancer Biology*	U.K.	30	20.3	134	Q1
*Frontiers in Cellular and Infection Microbiology*	Switzerland	28	5.5	53	Q1

**Figure 6:**
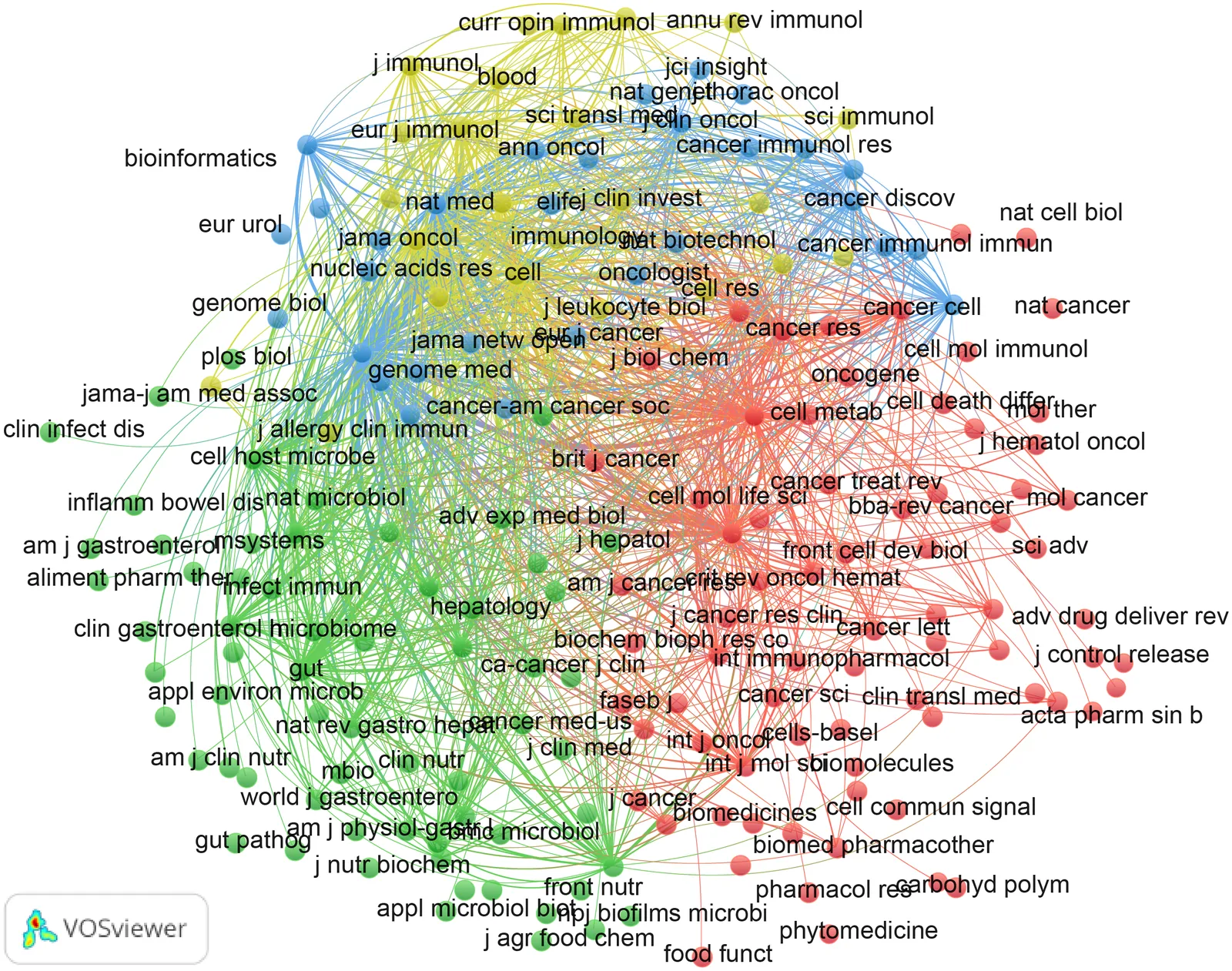
Journal co-citation network in cancer immunotherapy and gut microbiota metabolism research. Nodes represent journals. The network was generated using VOSviewer.

Co-citation analysis of journals using CiteSpace (**Figure 6**) revealed that Alimentary Pharmacology & Therapeutics (centrality = 0.17), American Journal of Clinical Nutrition (0.04), and Nature (0.03) ranked highest in terms of centrality (**Supplementary Table 5**). Furthermore, a dual-map overlay of citing and cited journals generated using CiteSpace was provided as **Supplementary Figure 1**, highlighting the interdisciplinary knowledge flow between basic molecular sciences and clinical medicine in cancer immunotherapy and gut microbiota metabolism research.

Among original articles, 84 journals published at least five articles, accounting for 705 publications (50.87% of all original articles). Frontiers in Immunology remained the most productive journal, with 88 original articles (**Supplementary Table 7**), supporting the robustness of the overall journal-level publication pattern.

### 3.6 Keyword co-occurrence network

Keyword frequency analysis (**Table 3**) showed that, excluding the primary search terms (e.g., immunotherapy, cancer, ICIs, gut microbiota, and microbiome), the most frequent keywords included tumor microenvironment (121), colorectal cancer (94), probiotics (80), inflammation (51), and dysbiosis (49).

**Table 3:** Top ten high-frequency keywords and keywords with the highest centrality coefficient.

Rank	Keywords	Frequency	Keywords	Centrality
1	immunotherapy	398	*Akkermansia muciniphila*	0.09
2	gut microbiota	216	Short-chain fatty acids	0.08
3	cancer	149	CD8+ T cells	0.08
4	microbiome	136	*Fusobacterium nucleatum*	0.07
5	tumor microenvironment	121	dendritic cells	0.07
6	colorectal cancer	94	bacteria	0.06
7	ICIs	93	expression	0.06
8	probiotics	80	activation	0.06
9	inflammation	51	suppressor cells	0.06
10	dysbiosis	49	*Clostridium difficile infection*	0.06

Keywords with the highest centrality included Akkermansia muciniphila (0.09), short-chain fatty acids (0.08), and CD8+ T cells (0.08).

**Figure 7:**
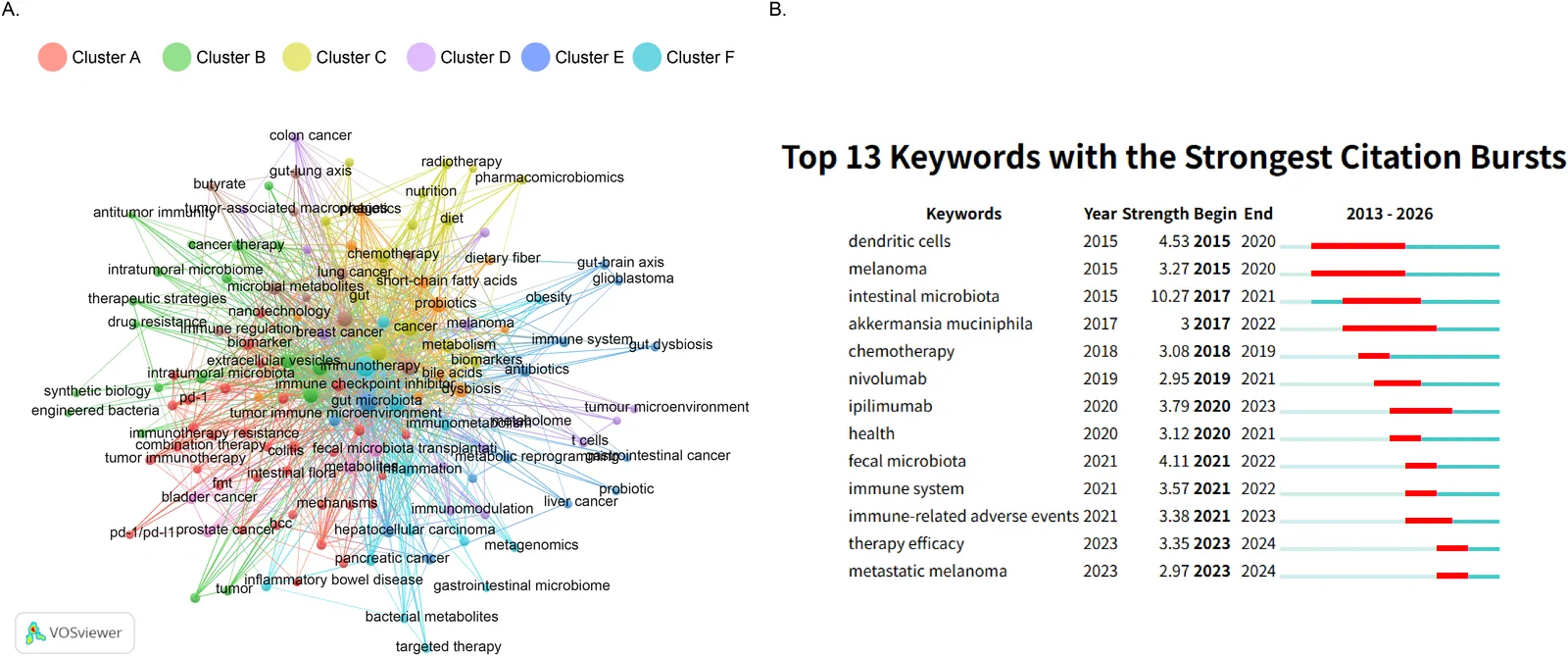
Keyword co-occurrence network and burst analysis in cancer immunotherapy and gut microbiota metabolism research. **(A)** Keyword co-occurrence network generated using VOSviewer. **(B)** Keyword citation burst analysis generated using CiteSpace.

Further keyword co-occurrence analysis identified six major clusters (**Figure 7A**). The red cluster focuses on immunotherapy resistance and combinatorial strategies, featuring immunotherapy resistance, PD-1/PD-L1 inhibitors, fecal microbiota transplantation, and combination therapy. The green cluster centers on microbiota-mediated antitumor immunity and tumor microenvironment regulation, including intratumoral microbiota, tumor-associated macrophages, antitumor immunity, and dendritic cells. The yellow cluster highlights microbial metabolites and dietary interventions, such as short-chain fatty acids, butyrate, probiotics, diet, and nutrition. The purple cluster addresses gut dysbiosis, inflammation, and metabolic disorders, covering dysbiosis, inflammation, metabolic reprogramming, and colon cancer. The blue cluster concentrates on clinical translation and multi-omics in gastrointestinal cancers, including the gastrointestinal microbiome, hepatocellular carcinoma, metabolomics, and metagenomics. The cyan cluster explores ICIs and clinical outcomes, involving nivolumab, ipilimumab, melanoma, chemotherapy, and immune-related adverse events.

Keyword burst detection analysis (**Figure 7B**) revealed that intestinal microbiota had the strongest burst intensity (strength = 10.27), followed by dendritic cells (4.53) and fecal microbiota (4.11).

Sensitivity analysis restricted to original articles showed that the overall keyword landscape was broadly consistent with that of the full dataset. The most frequent keywords were immunotherapy (124), gut microbiota (80), colorectal cancer (35), tumor microenvironment (33), and gut microbiome (32), while gut microbiota (0.20) and immunotherapy (0.16) showed the highest centrality values (**Supplementary Table 8**).

### 3.7 Research trends

The temporal evolution of keywords provides insights into the development trajectory of this field. Based on median year analysis using the bibliometrix package (**Table 4**), the research can be broadly divided into several phases. Before 2016, the field was at an early stage with limited publications and no clearly defined hotspots. Early keywords such as single-chain variable fragment, food allergy, and ulcerative colitis suggest a focus on basic immunology and inflammatory diseases. Between 2017 and 2019, research began to transition toward immunotherapy-related topics, with increased attention to phage display, checkpoint inhibitors, dendritic cells, cancer vaccines, and interferon-γ (IFN-γ), reflecting a shift from basic mechanisms to disease-oriented studies. From 2020 onward, the research focus shifted markedly toward cancer immunotherapy and its clinical determinants. Keywords such as PD-1, CTLA-4, antibiotics, inflammation, and biomarkers highlight increasing interest in factors influencing therapeutic efficacy. Meanwhile, the emergence of microbiome-related terms indicates growing recognition of the interaction between gut microbiota and cancer immunotherapy. Since 2023, the field has entered a phase of rapid development, with immunotherapy, gut microbiota, and tumor microenvironment becoming dominant research themes. More recently (2025–present), emerging hotspots include immunomodulation, the gut–liver axis, and intratumoral microbiota. Notably, intratumoral microbiota, with a median year extending to 2026, represents a cutting-edge research frontier.

**Table 4:** Keyword time zone for research related to cancer immunotherapy and intestinal flora metabolism.

Term	Frequency	Year (Q1)	Year (Median)	Year (Q3)
Single-chain Variable Fragment	7	2016	2016	2018
food allergy	5	2014	2016	2019
ulcerative colitis	5	2015	2016	2020
phage display	5	2016	2017	2020
checkpoint inhibitors	6	2018	2018	2020
crohn’s disease	5	2016	2018	2020
dendritic cells	9	2015	2019	2021
cancer vaccine	6	2014	2019	2024
IFN-γ	5	2017	2019	2020
inflammation	44	2018	2020	2023
antibiotics	17	2020	2020	2022
CTLA-4	13	2020	2020	2021
PD-1	37	2020	2021	2023
biomarkers	31	2020	2021	2024
immune system	17	2019	2021	2024
cancer	115	2020	2022	2024
microbiome	95	2020	2022	2025
microbiota	89	2020	2022	2023
immunotherapy	398	2022	2023	2025
gut microbiota	216	2022	2024	2025
tumor microenvironment	121	2022	2024	2025
microbial metabolites	23	2024	2025	2025
immunomodulation	21	2023	2025	2025
gut-liver axis	14	2022	2025	2025
intratumoral microbiota	11	2026	2026	2026

**Figure 8:**
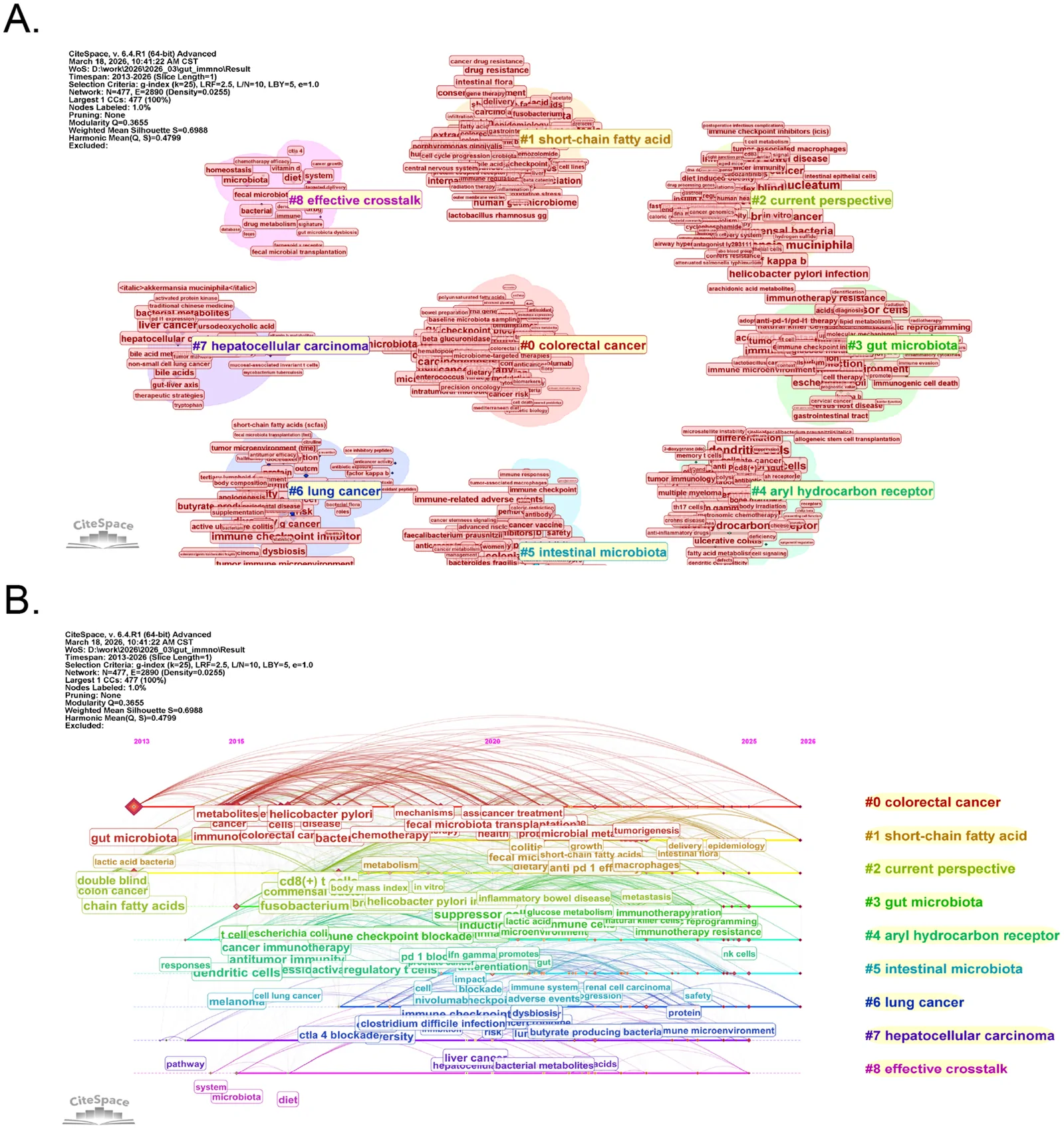
Temporal evolution of research keywords in cancer immunotherapy and gut microbiota metabolism research. Generated using CiteSpace. **(A)** Keyword clusters. **(B)** Timeline visualization of keyword evolution.

CiteSpace clustering of timeline keywords (**Figure 8A**) identified major clusters including colorectal cancer, short-chain fatty acids, aryl hydrocarbon receptor, intestinal microbiota, lung cancer, and hepatocellular carcinoma. Research on colorectal cancer and hepatocellular carcinoma spanned from 2013 to 2026, while lung cancer-related studies were primarily concentrated between 2015 and 2026. Additionally, short-chain fatty acids and the aryl hydrocarbon receptor have consistently attracted attention as key metabolic-related markers (**Figure 8B**).

Sensitivity analysis restricted to original articles showed that lung cancer and hepatocellular carcinoma remained prominent cancer-related research themes, while intratumoral microbiota continued to emerge as an important research focus (**Supplementary Figure 2**), supporting the robustness of the major research trends identified in the overall dataset.

### 3.8 Reference burst analysis

**Figure 9:**
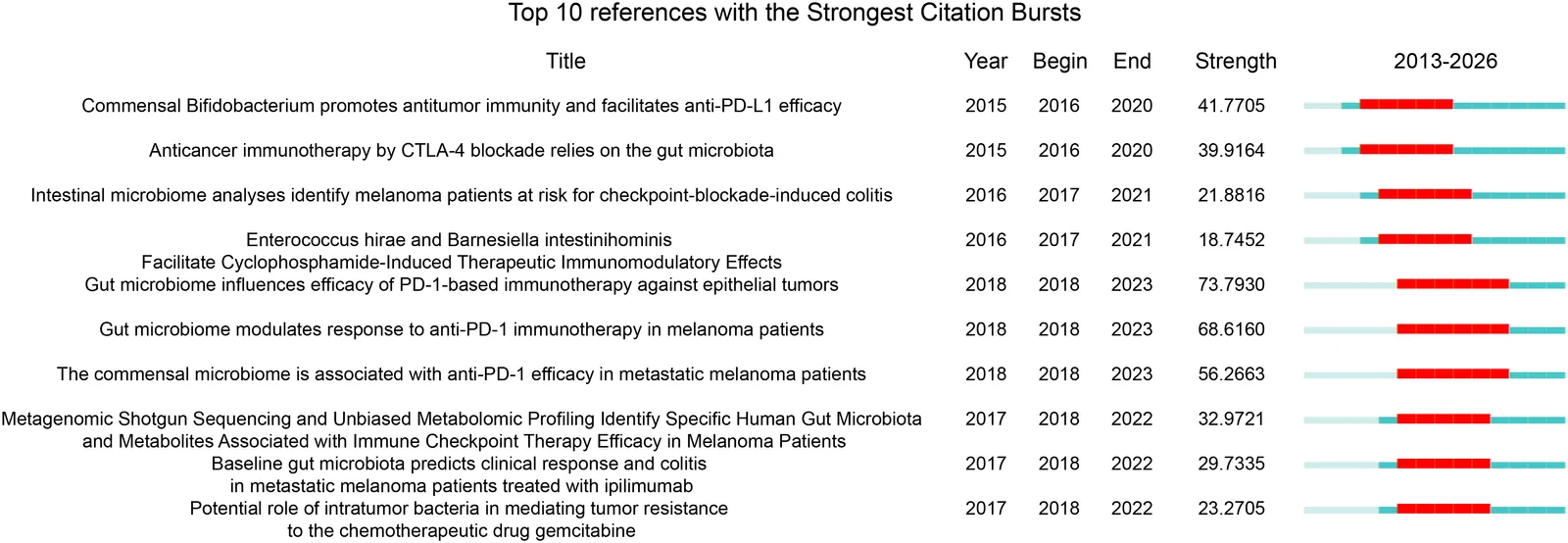
Top 10 references with the strongest citation bursts in cancer immunotherapy and gut microbiota metabolism research.

Reference burst analysis further reflected shifts in research focus within this field (**Figure 9**). The publication with the strongest citation burst was “Gut microbiome influences efficacy of PD-1-based immunotherapy against epithelial tumors” (strength = 73.7930), which demonstrated that primary resistance to PD-1 blockade is associated with gut microbiota composition in patients with epithelial tumors (Routy et al., 2018).

This was followed by “Gut microbiome modulates response to anti-PD-1 immunotherapy in melanoma patients” (strength = 68.6160), which demonstrated an association between gut microbiota composition and response to anti–PD-1 immunotherapy in melanoma patients (Gopalakrishnan et al., 2018), and “The commensal microbiome is associated with anti-PD-1 efficacy in metastatic melanoma patients” (strength = 56.2663), which suggested that commensal microbiota may modulate antitumor immune responses in metastatic melanoma (Matson et al., 2018).

Notably, although these highly cited studies primarily focused on microbiota composition, they have laid the foundation for subsequent research exploring functional mechanisms, particularly the role of gut microbiota-derived metabolites in modulating immunotherapy responses.

## 4 Discussion

### 4.1 General trends and research evolution

In this bibliometric analysis, a total of 2,979 publications published between 1973 and March 2026 were identified, reflecting sustained and increasing research interest in this field. The annual publication trend showed a prolonged period of relatively slow growth prior to 2019, followed by a marked and rapid increase thereafter, indicating an accelerated development phase in recent years. This rapid expansion likely reflects the increasing clinical importance of cancer immunotherapy, particularly immune checkpoint blockade, together with growing recognition of the gut microbiota and its functional metabolites as important regulators of therapeutic responses.

Globally, research contributions have been widely distributed across 75 countries and regions, with China and the United States emerging as the leading contributors. While China accounts for the largest number of publications, the United States demonstrates greater academic impact in terms of H-index and citation counts, suggesting a structural difference between research productivity and influence. This discrepancy may reflect differences in research maturity, scientific infrastructure, and international collaboration. The United States entered this field earlier and has contributed to several landmark studies on immune checkpoint blockade and translational immunotherapy, which may explain its higher citation impact. By contrast, China’s rapid increase in publication output likely reflects substantial investment in biomedical research and growing interest in microbiome-based therapeutic strategies, although its comparatively shorter research history and developing international collaboration networks may have limited its citation influence. The original-article-only sensitivity analysis further supported the robustness of the observed country-level research pattern.

### 4.2 Global research patterns and collaboration

Analysis of collaboration networks revealed notable disparities between research productivity and international influence. At the country level, the United States and the United Kingdom occupied central positions within the global collaboration network, whereas China ranked relatively lower in terms of centrality. This suggests that although China has achieved substantial research output, its role in international collaboration networks is comparatively less prominent than that of Western countries. This pattern suggests that international collaboration remains an important determinant of global scientific influence and may partly explain the disparity between publication output and citation impact observed across countries. The original-article-only sensitivity analysis further supported the robustness of the observed international collaboration structure.

At the institutional level, major collaboration hubs were formed around the University of Texas System, The University of Texas MD Anderson Cancer Center, and the Institut National de la Santé et de la Recherche Médicale (INSERM). Notably, six of the top ten most productive institutions were from China. In the overall dataset including both original articles and reviews, Sichuan University ranked first in publication output. Representative studies from Sichuan University have further highlighted the therapeutic potential of gut microbiota-targeted interventions as adjunctive strategies to enhance immune cell-based therapies in solid tumors (Hu et al., 2025). However, sensitivity analysis restricted to original articles showed that Shanghai Jiao Tong University became the leading institution, followed by Fudan University and the Chinese Academy of Sciences. These findings suggest that document type may influence institutional rankings, and that overall publication productivity does not necessarily reflect primary research contributions.

In terms of individual contributions, Laurence Zitvogel from INSERM was the most prolific author in this field. In a recent review, Zitvogel and colleagues highlighted the association between gut microbiota dysbiosis and tumor immune resistance, proposing that microbiome-based therapeutic strategies may offer novel treatment opportunities for patients who are unresponsive to immunotherapy (Almonte et al., 2026).

### 4.3 Mechanistic insights and emerging research themes

Based on keyword co-occurrence and citation burst analyses, a clear evolution in research focus was identified. Early studies primarily emphasized associations between gut microbiota composition and immunotherapy resistance, whereas recent research has shifted toward a functional paradigm centered on gut microbiota-derived metabolites and systemic regulatory pathways. These findings indicate that the research landscape has expanded from taxonomic and descriptive questions toward functional and system-level topics, with “microbial metabolites”, “immunomodulation”, “the gut–liver axis”, and “intratumoral microbiota” emerging as prominent themes. The original-article-only sensitivity analysis further supported the robustness of the major research themes identified in the overall dataset.

#### 4.3.1 The gut–liver axis as an emerging research hotspot

Based on the keyword co-occurrence and citation burst analyses, the gut–liver axis has emerged as an important research hotspot in the field of cancer immunotherapy. This emerging trend reflects growing research interest in the possibility that gut microbiota may influence therapeutic responses not only through local intestinal mechanisms but also through systemic metabolic and immune communication. Through the portal circulation and immune signaling pathways, gut-derived microbial components can modulate hepatic immune homeostasis and broader host immune responses, thereby establishing a functional connection between intestinal microbial activity and systemic immunity (Fousekis et al., 2025).

From a biological perspective, the gut–liver axis represents a bidirectional regulatory network integrating intestinal barrier integrity, microbial-derived signals, hepatic metabolism, and immune regulation. Disruption of intestinal homeostasis facilitates the translocation of microbial-associated molecular patterns and microbial-derived molecules through the portal circulation to the liver, where they are sensed by hepatic immune and metabolic systems. Activation of pattern-recognition receptor pathways, including Toll-like receptor, myeloid differentiation primary response 88, and nuclear factor kappa-B signaling, contributes to the regulation of hepatic inflammatory responses and cytokine production, thereby shaping local hepatic immune homeostasis and systemic immune status (Tilg et al., 2022; Pabst et al., 2023). Emerging evidence suggests that dysregulation of this axis may influence tumor progression, immune escape, and variability in immunotherapy response, highlighting the gut–liver axis as an important framework for understanding microbiota-mediated regulation of cancer immunity (Pallozzi et al., 2024; Yang et al., 2025).

Among the metabolic factors involved in gut–liver communication, microbiota-associated metabolites, particularly bile acids, have attracted increasing attention as important mediators linking intestinal microbial activity with hepatic and systemic immune regulation (Duan et al., 2025; Tong and Lou, 2025). Emerging clinical and translational evidence further supports the role of bile acid–mediated and microbiota-driven systemic immune modulation in influencing responses to immune checkpoint inhibitors (Huang et al., 2025). Microbial modification of bile acid metabolism may influence host immune homeostasis and may contribute to heterogeneity in immunotherapy responses, suggesting a potential link between microbiota-driven metabolic signaling and therapeutic outcomes.

Collectively, the available evidence suggests that immunotherapy response may be influenced not only by local tumor factors but also by systemic microbiota-associated pathways, with the gut–liver axis representing a potential framework linking intestinal microbial activity to host immune responses. The emergence of the gut–liver axis in the bibliometric analysis therefore likely reflects a growing recognition that systemic metabolic communication, rather than local microbial composition alone, contributes to therapeutic heterogeneity and represents an important direction for translational research.

Despite growing interest in this area, current evidence is still largely derived from observational studies and preclinical models, and the causal relationships linking gut microbiota, bile acid signaling, and immunotherapy efficacy remain incompletely understood. Future research should focus on integrating microbiome, metabolome, and immune profiling data to identify predictive biomarkers and clarify the mechanistic basis of gut–liver axis-mediated regulation of cancer immunotherapy.

#### 4.3.2 Tumor microenvironment and intratumoral microbiota

Intratumoral microbiota has recently gained increasing attention as an emerging research focus in this field. This concept refers to the presence of microbial communities within tumor tissues that are distinct from gut microbiota and are typically characterized by low microbial biomass but functional activity within the tumor microenvironment, with the potential to influence cancer initiation, progression, and therapeutic responses (Galeano Niño et al., 2022; Yang et al., 2023). Unlike gut microbiota, which primarily regulate host immunity through systemic mechanisms, intratumoral microbiota provide a direct interface between microbial signals and the tumor microenvironment. Owing to their localization within tumor tissues, these microorganisms can directly interact with tumor cells and infiltrating immune cells, thereby exerting localized immunoregulatory effects.

Current evidence suggests that these microorganisms may modulate the tumor immune microenvironment through multiple pathways, including microbial-associated molecular signals and direct microbe–host interactions, thereby affecting immune surveillance within tumors (Liu et al., 2026). Emerging studies further indicate that intratumoral microbes can influence immune cell composition and function by regulating antigen presentation, macrophage polarization, and T cell activation or exhaustion states, potentially shaping antitumor immune responses in a context-dependent manner (Dohlman et al., 2021).

Beyond immune regulation, intratumoral microorganisms may influence tumor progression and therapeutic outcomes through metabolic interactions with cancer cells and anticancer agents. Certain bacteria residing within tumors have been shown to modify local drug metabolism, degrade or activate therapeutic compounds, and alter the intratumoral distribution or efficacy of chemotherapeutic agents (Xie et al., 2025). Such microbial-mediated metabolic activities have been associated with differential responses to anticancer therapies across multiple tumor types, highlighting intratumoral microbiota as a potential determinant of treatment sensitivity and resistance (Zhai et al., 2026). Furthermore, engineered intratumoral microorganisms have been explored as potential delivery platforms for anticancer agents, providing new avenues for precision cancer therapy (Zhai et al., 2026).

However, despite these advances, the functional role of intratumoral microbiota remains a subject of ongoing debate. One major challenge lies in distinguishing true tumor-resident microbes from potential contamination introduced during sample collection, sequencing, or analysis, particularly given the low microbial biomass in tumor tissues. In addition, current evidence is largely based on associative studies, and causal relationships between intratumoral microbiota and therapeutic outcomes have yet to be fully established.

Importantly, intratumoral microbiota do not function in isolation but are closely linked to gut microbiota and systemic microbial metabolism. Increasing evidence suggests that gut microbiota-derived metabolites originating from the gut can influence tumor-resident microbes or the tumor microenvironment via systemic circulation, highlighting a multi-scale regulatory network connecting gut microbiota, circulating metabolites, and intratumoral microbial ecosystems.

This cross-scale interaction suggests a conceptual framework in which gut microbiota, circulating metabolites, and intratumoral microorganisms collectively shape tumor immunity through coordinated local and systemic regulation. This conceptual framework further supports the bibliometric observation that research in this field is shifting from isolated investigations of microbial composition toward integrated analyses of microbial ecosystems and host regulatory networks.

Collectively, these findings indicate that intratumoral microbiota function as active regulators of the tumor microenvironment and represent an important component of integrated microbiota–host regulatory networks. Although intratumoral microbiota has only recently emerged as a bibliometric hotspot, its increasing integration with studies of tumor metabolism, spatial biology, and immunotherapy suggests that this topic is evolving beyond an exploratory stage. The convergence of continued research activity with accumulating mechanistic and translational evidence suggests that intratumoral microbiota may represent a sustained research direction rather than a transient research interest.

Future studies employing spatial transcriptomics, spatial metabolomics, and single-cell sequencing technologies may provide deeper insights into microbe–host interactions within tumor tissues. In addition, the development of standardized protocols for microbial detection and validation will be essential for translating intratumoral microbiota research into clinical applications.

#### 4.3.3 Gut microbiota-derived metabolites as key mediators of immunotherapy response

Consistent with the keyword co-occurrence and burst analyses, gut microbiota-derived metabolites, particularly SCFAs and aryl hydrocarbon receptor (AhR)-related pathways, emerged as one of the most active and rapidly expanding research themes. Beyond their increasing prominence in publication trends, accumulating mechanistic evidence indicates that these metabolites function as active signaling molecules rather than passive by-products, regulating immune responses through epigenetic modification, nuclear receptor activation, and metabolic reprogramming, which may contribute to differences in immunotherapy responses (Luo et al., 2025).

SCFAs, as major metabolic products of gut microbiota, have been implicated in diverse immunomodulatory processes, with context-dependent effects observed across different cancer types (Xiang et al., 2026). At the molecular level, SCFAs can influence immune responses through epigenetic regulation, particularly via inhibition of histone deacetylases (HDACs), thereby altering gene transcription programs associated with T cell activation and effector function. Notably, butyrate has been reported to enhance the efficacy of anti–PD-1 therapy by increasing histone H3K27 acetylation at the promoter regions of immune-related genes (e.g., Pdcd1 and Cd28), thereby promoting T cell receptor signaling and cytokine production in preclinical tumor models (Zhu et al., 2023). Importantly, the immunomodulatory effects of SCFAs appear to be highly context-dependent and may exhibit bidirectional roles in tumor immunity. At physiological concentrations, SCFAs may enhance effector T cell responses and antitumor immunity, whereas under certain tumor microenvironment conditions or at higher concentrations, they may promote regulatory T cell expansion and immune suppression. This dose- and context-dependent behavior highlights a complex regulatory mechanism that remains to be fully elucidated. At the clinical level, fecal SCFA levels, as well as the abundance of SCFA-producing bacteria (e.g., Faecalibacterium and Roseburia), are increasingly being explored as potential biomarkers for predicting responses to immune checkpoint inhibitors across multiple cancer types (Xiang et al., 2026).

As an important metabolic component of the gut–liver axis discussed above, bile acids represent a major class of microbiota-derived metabolites that function as immunological mediators connecting microbial activity with host immune regulation. Through microbial remodeling of the bile acid pool, gut microorganisms generate diverse secondary bile acid species that act as signaling molecules through host receptors including the farnesoid X receptor (FXR) and Takeda G protein-coupled receptor 5 (TGR5) (Duan et al., 2025; Tong and Lou, 2025). These metabolite-mediated signaling pathways regulate immune cell differentiation, inflammatory responses, and immune metabolic states within the tumor microenvironment. Emerging evidence indicates that specific secondary bile acid profiles are associated with enhanced antitumor immunity and improved responses to immune checkpoint inhibitors, potentially through modulation of CD8^+^ T-cell function, dendritic cell activation, and myeloid cell polarization (Huang et al., 2025). Collectively, these findings provide a plausible mechanistic link between microbiota-driven bile acid metabolism and systemic immune regulation, and support further investigation of bile acid signatures as potential biomarkers or therapeutic targets in cancer immunotherapy.

In parallel, tryptophan-derived microbial metabolites have attracted increasing attention through activation of the AhR, a ligand-activated transcription factor with broad immunoregulatory functions. Activation of AhR by microbiota-derived ligands, particularly tryptophan metabolites, has been shown to regulate immune homeostasis by modulating the balance between effector T cells and regulatory T cells, thereby shaping the tumor immune microenvironment. Emerging evidence further suggests that modulation of AhR signaling may influence tumor progression and responsiveness to immune checkpoint blockade, supporting its potential as a therapeutic target (Perdew et al., 2023). Notably, AhR signaling may serve as a convergence node for multiple microbiota-derived metabolic pathways, integrating tryptophan metabolism with broader immune regulatory networks and coordinating context-dependent immune responses across different tumor types (Hezaveh et al., 2022).

These mechanisms involve multiple, potentially interconnected pathways, including epigenetic regulation (e.g., HDAC inhibition), receptor-mediated signaling (e.g., GPCRs, FXR, TGR5, and AhR), and metabolic reprogramming, through which gut microbiota-derived metabolites may influence immune cell differentiation, cytokine production, and immune checkpoint signaling.

Taken together, these findings support a multi-level conceptual framework in which gut microbiota-derived metabolites may link microbial activity with host immune regulation across multiple biological scales. At the molecular level, available evidence suggests that metabolites can affect gene expression and signaling pathways; at the cellular level, they may influence immune cell differentiation and functional states; and at the systemic level, they may contribute to inter-organ communication through pathways such as the gut–liver axis. This multi-scale perspective provides a useful biological context for understanding the growing research interest in microbial metabolites and their potential relevance to heterogeneity in immunotherapy responses.

However, despite growing evidence supporting their functional importance, several challenges remain. The context-dependent effects of gut microbiota-derived metabolites, variability across tumor types, and inter-individual differences in microbiota composition complicate their translational application. In addition, most current studies are associative, and further mechanistic and interventional investigations are required to establish causal relationships.

Looking forward, gut microbiota-derived metabolites are likely to remain a major research direction in this field, supported by sustained growth in keyword occurrence and accumulating mechanistic evidence. Future studies should prioritize causal validation of metabolite-mediated pathways, quantitative characterization of metabolite–immune interactions, and the development of metabolite-based biomarkers and therapeutic interventions. Integrating bibliometric findings with current biological evidence suggests that research in cancer immunotherapy is increasingly shifting from descriptive microbial profiling toward mechanism-oriented investigations centered on gut microbiota-derived metabolites and systemic regulatory networks, providing new opportunities for precision biomarkers and microbiota-targeted therapeutic strategies.

### 4.4 Challenges and future perspectives

Collectively, these findings suggest that increasing research attention is being directed toward the functional activities of the microbiome, including metabolite production, host–microbe signaling, and gut microbiota-derived metabolites, rather than microbial composition alone. These topics may provide complementary perspectives for understanding variability in cancer immunotherapy responses. These themes span metabolic regulation, immune modulation, systemic communication, and tumor-localized microbial interactions. Their co-occurrence in the literature suggests increasing interest in understanding these processes in an integrated manner, although the biological relationships among them require further experimental validation. Rather than acting independently, emerging research hotspots—including “microbial metabolites”, “the gut–liver axis”, and “intratumoral microbiota”—can be conceptualized as interconnected components of an integrated regulatory framework spanning molecular, systemic, and tumor-localized levels.

However, several challenges remain. First, methodological heterogeneity continues to limit the comparability and reproducibility of current findings. Variations in patient populations, tumor types, treatment regimens, antibiotic exposure, and microbiome sequencing methodologies may lead to inconsistent results. In addition, differences in metabolomic profiling techniques and analytical pipelines further complicate cross-study comparisons. Second, biological complexity and context dependency present major obstacles to mechanistic interpretation. The effects of microbiota and their metabolites are highly dependent on tumor type, host immune status, and environmental factors, resulting in heterogeneous and sometimes contradictory findings. Third, technical limitations remain particularly prominent in the study of intratumoral microbiota. Issues such as low microbial biomass, contamination risk, and limited sensitivity of detection methods continue to hinder reproducibility and data interpretation. Finally, the lack of causal evidence represents a critical gap in the field. Most current mechanistic insights are derived from preclinical or associative studies, and robust causal validation in clinical settings remains insufficient.

From a translational perspective, the identified research themes point to several areas of potential clinical interest. Gut microbiota-derived metabolites are being investigated as potential biomarkers of immunotherapy response. Furthermore, microbiome-targeted interventions—including dietary modulation, probiotics, fecal microbiota transplantation, and metabolite-based approaches—are being explored as strategies to potentially enhance immunotherapy efficacy. However, their clinical implementation remains challenging due to issues related to safety, standardization, inter-individual variability, and regulatory considerations, highlighting the need for well-designed clinical trials.

Future research should focus on integrating multi-omics approaches, including metagenomics, metabolomics, and transcriptomics, to comprehensively characterize microbiota–host interactions across different biological layers. Longitudinal and interventional studies will be essential to capture dynamic changes in the microbiome during immunotherapy and to establish causal relationships. In addition, the development of standardized analytical frameworks and validated biomarkers will be critical to improve reproducibility and facilitate clinical translation.Importantly, future studies should also focus on identifying key functional metabolites and their target pathways, which may enable the development of more precise microbiome-based therapeutic strategies.

### 4.5 Limitations

Several limitations should be acknowledged in this study. First, although WoSCC and Scopus are among the most widely used databases for bibliometric studies, the exclusion of other databases may have resulted in the omission of relevant publications. Differences in database coverage and indexing policies may also introduce potential selection bias. Second, only English-language publications were included, which may introduce language bias, particularly because a considerable proportion of research output originates from non-English-speaking countries. Future studies incorporating non-English literature may provide a more comprehensive perspective. Third, the bibliometric software tools used in this study are subject to continuous updates, and variations in software versions may lead to minor discrepancies in analytical results. Fourth, bibliometric indicators such as citation counts and H-index may not fully reflect the true scientific impact of publications, as they can be influenced by factors such as publication time, citation practices, and disciplinary differences. Fifth, due to the dynamic nature of scientific research, recently published studies may be underrepresented in citation-based analyses, resulting in a potential time-lag bias. Sixth, the inclusion of both original articles and review papers in the overall bibliometric analysis may influence institutional productivity rankings, as institutions with substantial contributions to review literature may appear disproportionately influential. To address this issue, an additional sensitivity analysis restricted to original articles was performed. Although certain institutional rankings changed, the overall research landscape and collaboration patterns remained largely consistent, supporting the robustness of the present findings.

## 5 Conclusions

In summary, this bibliometric analysis highlights a rapidly evolving research landscape in which both China and the United States play leading roles in advancing this field. Importantly, the field is undergoing a paradigm shift from descriptive microbiome profiling toward functional and mechanism-oriented research, with gut microbiota-derived metabolites emerging as prominent mechanistic mediators investigated in relation to cancer immunotherapy responses. Emerging research themes—including “microbial metabolites”, “the gut–liver axis”, and “intratumoral microbiota”—collectively represent a multi-scale framework that integrates molecular, local, and systemic regulation of tumor immunity. These advances underscore the critical role of microbiota–host metabolic crosstalk in shaping antitumor immune responses and determining therapeutic outcomes. Future research should prioritize causal validation of metabolite-mediated pathways, integration of multi-omics data (including microbiome and metabolome), and the establishment of standardized analytical frameworks to enhance reproducibility and translational applicability. Ultimately, a deeper understanding of microbiota–metabolite–host interactions may facilitate the development of targeted metabolic interventions, paving the way for more precise and effective cancer immunotherapy strategies.

## Data Availability

The original contributions presented in the study are included in the article/**Supplementary Material**. Further inquiries can be directed to the corresponding author.

## Supplementary material

Supplementary Figure 1 Dual-map overlay of citing and cited journals generated using CiteSpace, illustrating citation trajectories across research disciplines.

Supplementary Figure 2 Keyword timeline analysis of original articles on cancer immunotherapy and gut microbiota metabolism, generated using CiteSpace. **(A)** Keyword cluster visualization; **(B)** Keyword timeline visualization.
