# Supplementary material for: Global trends and emerging frontiers in cancer immunotherapy and gut microbiota-derived metabolites: a bibliometric analysis
Source: Front Cell Infect Microbiol. 2026 Sep 3;16:1876450. doi: 10.3389/fcimb.2026.1876450 (PMC13581584; doi:10.3389/fcimb.2026.1876450)
Supplement: Supplementary file 1 [file Table1.docx]

**Supplementary Table 1.** Detailed search strategies for Web of Science Core Collection and Scopus databases

| **Database** | **Search strategy** | | **Result** |
| --- | --- | --- | --- |
| WoSCC | #1 | (“immunotherapy” OR “immune checkpoint inhibitor” OR “immune checkpoint blockade” OR “chimeric antigen receptor”) AND (“cancer” OR “tumor” OR “tumour” OR “neoplasm*” OR “oncology”) (Topic) | 1084 |
|  | #2 | (“microbiome” OR “microflora” OR “microbiota” OR “flora” OR “probiotic” OR “Saccharomyces” OR “Lactobacillus” OR “Bifidobacterium” OR “Escherichia coli”) AND (“metabolite*” OR “metabolism” OR “metabolome” OR “bile acids” OR “primary bile acids” OR “secondary bile acids” OR “bile salts” OR “conjugated bile acids” OR “short-chain fatty acids” OR “SCFAs” OR “acetate” OR “propionate” OR “butyrate” OR “tryptophan” OR “neurotransmitters” OR “GABA” OR “dopamine” OR “5-HT” OR “serotonin” OR “vitamins” OR “vitamin D” OR “vitamin B6” OR “hypoxanthine” OR “fatty acids” OR “amino acids” OR “ammonia” OR “amines” OR “phenols” OR “indoles”) (Topic) |  |
|  | #3 | #1 AND #2 AND Document Types: Article or Review Article AND Languages: English |  |
| Scopus | #1 | TITLE-ABS-KEY (“immunotherapy” OR “immune checkpoint inhibitor” OR “immune checkpoint blockade” OR “chimeric antigen receptor”) AND (“cancer” OR “tumor” OR “tumour” OR “neoplasm*” OR “oncology”) | 2724 |
|  | #2 | TITLE-ABS-KEY (“microbiome” OR “microflora” OR “microbiota” OR “flora” OR “probiotic” OR “Saccharomyces” OR “Lactobacillus” OR “Bifidobacterium” OR “Escherichia coli”) AND (“metabolite*” OR “metabolism” OR “metabolome” OR “bile acids” OR “primary bile acids” OR “secondary bile acids” OR “bile salts” OR “conjugated bile acids” OR “short-chain fatty acids” OR “SCFAs” OR “acetate” OR “propionate” OR “butyrate” OR “tryptophan” OR “neurotransmitters” OR “GABA” OR “dopamine” OR “5-HT” OR “serotonin” OR “vitamins” OR “vitamin D” OR “vitamin B6” OR “hypoxanthine” OR “fatty acids” OR “amino acids” OR “ammonia” OR “amines” OR “phenols” OR “indoles”) |  |
|  | #3 | #1 AND #2 AND (LIMIT-TO ( DOCTYPE,"ar" ) OR LIMIT-TO (DOCTYPE,"re")) AND (LIMIT-TO (LANGUAGE,"English")) |  |
