# Supplementary material for: Global trends and emerging frontiers in cancer immunotherapy and gut microbiota-derived metabolites: a bibliometric analysis
Source: Front Cell Infect Microbiol. 2026 Sep 3;16:1876450. doi: 10.3389/fcimb.2026.1876450 (PMC13581584; doi:10.3389/fcimb.2026.1876450)
Supplement: Supplementary file 2 [file Table3.docx]

**Supplementary Table 3.** Keyword thesaurus/harmonization list

| **Keywords** | **synonym** |
| --- | --- |
| SCFAs | short-chain fatty acids, short chain fatty acids, SCFA |
| IFN-γ | Interferon gamma, Interferon-γ, IFN-gamma, gamma-interferon, gamma interferon |
| CTLA-4 | Cytotoxic T-lymphocyte-associated antigen 4, Cytotoxic T-lymphocyte-associated protein 4, CTLA4, CTLA 4 |
| PD-1 | Programmed cell death 1, Programmed cell death protein 1, Programmed Death-1, PDCD1 |
| AhR | Aryl hydrocarbon receptor, Arylhydrocarbon receptor, Aromatic hydrocarbon receptor, Ah receptor, AHR |
