# Supplementary material for: Global trends and emerging frontiers in cancer immunotherapy and gut microbiota-derived metabolites: a bibliometric analysis
Source: Front Cell Infect Microbiol. 2026 Sep 3;16:1876450. doi: 10.3389/fcimb.2026.1876450 (PMC13581584; doi:10.3389/fcimb.2026.1876450)
Supplement: Supplementary file 3 [file Table4.docx]

**Supplementary Table 4.** Publication output, H-index, and total citations of the top 10 countries based on original articles

| **Nation** | **Articles** | **H-index** | **Total citations** |
| --- | --- | --- | --- |
| China | 562 | 54 | 13996 |
| USA | 245 | 71 | 28527 |
| Japan | 59 | 23 | 1553 |
| Germany | 43 | 21 | 2009 |
| Iran | 38 | 12 | 366 |
| Italy | 36 | 17 | 2589 |
| Korea | 34 | 14 | 1211 |
| U.K. | 31 | 14 | 765 |
| France | 29 | 12 | 2158 |
| Canada | 28 | 9 | 2215 |
