# Supplementary material for: Global trends and emerging frontiers in cancer immunotherapy and gut microbiota-derived metabolites: a bibliometric analysis
Source: Front Cell Infect Microbiol. 2026 Sep 3;16:1876450. doi: 10.3389/fcimb.2026.1876450 (PMC13581584; doi:10.3389/fcimb.2026.1876450)
Supplement: Supplementary file 4 [file Table6.docx]

**Supplementary Table 6.** Top 10 institutions by publication count in non-review articles.

| **Institutions** | **Nation** | **Articles** |
| --- | --- | --- |
| Shanghai Jiao Tong University | China | 40 |
| Fudan University | China | 33 |
| Chinese Academy Of Sciences | China | 23 |
| Harvard Medical School | USA | 20 |
| Huazhong University Of Science And Technology | China | 20 |
| University Of California | USA | 18 |
| University Of Texas System | USA | 18 |
| Wuhan University | China | 17 |
| Institut National De La Sante Et De La Recherche Medicale (Inserm) | France | 16 |
| Tsinghua University | China | 16 |
