# Supplementary material for: Global trends and emerging frontiers in cancer immunotherapy and gut microbiota-derived metabolites: a bibliometric analysis
Source: Front Cell Infect Microbiol. 2026 Sep 3;16:1876450. doi: 10.3389/fcimb.2026.1876450 (PMC13581584; doi:10.3389/fcimb.2026.1876450)
Supplement: Supplementary file 5 [file Table7.docx]

**Supplementary Table 7.** Top 10 journals by the number of original articles on cancer immunotherapy and gut microbiota metabolism

| **Journal** | **Nation** | **Number of articles published** | **Impact factor（2025-2026）** | **H-index** | **JCR** |
| --- | --- | --- | --- | --- | --- |
| *Frontiers in Immunology* | Switzerland | 88 | 7.0 | 84 | Q1 |
| *Journal for ImmunoTherapy of Cancer* | USA | 44 | 10.6 | 36 | Q1 |
| *Nature Communications* | U.K. | 26 | 18.1 | 248 | Q1 |
| *Seminars in Cancer Biology* | U.K. | 18 | 15.7 | 134 | Q1 |
| *Scientific Reports* | U.K. | 16 | 4.9 | 149 | Q1 |
| *Biomaterials* | Netherlands | 15 | 13.6 | 337 | Q1 |
| *Cancer Discovery* | USA | 15 | 29.5 | 119 | Q1 |
| *Cell Reports Medicine* | USA | 15 | 14 | - | Q1 |
| *Gut Microbes* | USA | 15 | 15.3 | - | Q1 |
| *Journal of Translational Medicine* | U.K. | 15 | 9.7 | 87 | Q1 |
