# Supplementary material for: Global trends and emerging frontiers in cancer immunotherapy and gut microbiota-derived metabolites: a bibliometric analysis
Source: Front Cell Infect Microbiol. 2026 Sep 3;16:1876450. doi: 10.3389/fcimb.2026.1876450 (PMC13581584; doi:10.3389/fcimb.2026.1876450)
Supplement: Supplementary file 6 [file Table8.docx]

**Supplementary Table 8.** Top 10 keywords by frequency and centrality in original articles after excluding review articles

| **Rank** | **Keywords** | **Frequency** | **Keywords** | **Centrality** |
| --- | --- | --- | --- | --- |
| 1 | immunotherapy | 124 | gut microbiota | 0.2 |
| 2 | gut microbiota | 80 | immunotherapy | 0.16 |
| 3 | colorectal cancer | 35 | cancer immunotherapy | 0.14 |
| 4 | tumor microenvironment | 33 | efficacy | 0.12 |
| 5 | gut microbiome | 32 | activation | 0.12 |
| 6 | colorectal cancer | 25 | metabolites | 0.11 |
| 7 | immune checkpoint inhibitors | 22 | chain fatty acids | 0.11 |
| 8 | PD-L1 | 20 | responses | 0.11 |
| 9 | T cells | 19 | fusobacterium nucleatum | 0.09 |
| 10 | probiotics | 18 | T cells | 0.09 |
